# Laboratory Surveillance of Rabies in Humans, Domestic Animals, and Bats in Madagascar from 2005 to 2010

**DOI:** 10.4061/2011/727821

**Published:** 2011-08-21

**Authors:** Jean-Marc Reynes, Soa Fy Andriamandimby, Girard Marcelin Razafitrimo, Josette Razainirina, Elisabeth Marie Jeanmaire, Hervé Bourhy, Jean-Michel Heraud

**Affiliations:** ^1^National Laboratory for Rabies, Virology Unit, Institut Pasteur de Madagascar, Route de l'Institut Pasteur, BP 1274, 101 Antananarivo, Madagascar; ^2^Lyssavirus Dynamics and Host Adaptation Unit, National Reference Centre for Rabies, WHO Collaborating Centre for Reference and Research on Rabies, Institut Pasteur, 75724 Paris, France

## Abstract

*Background*. Rabies virus (RABV) has circulated in Madagascar at least since the 19th century. *Objectives*. To assess the circulation of lyssavirus in the island from 2005 to 2010. *Materials and Methods*. Animal (including bats) and human samples were tested for RABV and other lyssavirus using antigen, ribonucleic acid (RNA), and antibodies detection and virus isolation. *Results*. Half of the 437 domestic or tame wild terrestrial mammal brains tested were found RABV antigen positive, including 54% of the 341 dogs tested. This percentage ranged from 26% to 75% across the period. Nine of the 10 suspected human cases tested were laboratory confirmed. RABV circulation was confirmed in 34 of the 38 districts sampled. No lyssavirus RNA was detected in 1983 bats specimens. Nevertheless, antibodies against Lagos bat virus were detected in the sera of 12 among 50 *Eidolon dupreanum* specimens sampled. *Conclusion*. More than a century after the introduction of the vaccine, rabies still remains endemic in Madagascar.

## 1. Introduction

Rabies is a zoonotic disease caused by 11 viral species belonging to the genus *Lyssavirus* (Rhabdoviridae family), including the rabies virus (RABV), the most common [1–3]. These viruses are responsible for a meningoencephalomyelitis in mammals. Transmission of the viruses to a healthy mammal occurs mainly through bite or scratch by an infected mammal (the saliva is the infectious material). Bats are considered as the natural hosts of 10 of these viral species. However, dogs are the main source of infection in humans. It is estimated that 55,000 deaths per year worldwide are due to rabies infection with about 56% of which occur in Asia and 44% in Africa. In Africa and Asia, these deaths are responsible for 1.74 million disability-adjusted life years (DALYs) lost each year [4]. There is no effective treatment when the disease is declared. However, there is an effective treatment against RABV and closed related lyssaviruses when applied as soon as possible after exposure. It prevents the onset of symptom and death and consists of local treatment of the wound, administration of rabies immunoglobulin (if indicated), and vaccinations against rabies [5].

Lyssaviruses are present in all continents with the exception of Antarctica. RABV is the most widespread, widely distributed across the globe, with only a few countries (mainly islands and peninsulas) being free of the disease. Madagascar, an island in the south-western part of the Indian Ocean, does not belong to these exceptions (http://www.who.int/rabies/rabies_maps/en/index.html). Rabies virus has circulated in Madagascar at least since the 19th century. The son of one administrator of the former French Colony was reported dead of rabies in 1896, and his death was one of the reasons of the establishment of the Institut Pasteur in Madagascar in 1898. The first rabies postexposure treatment using rabies vaccine was implemented in 1902. Since that period, several reports have described the rabies situation in the island [6–9]. The last one, covering the 1982 through 1991 period, indicated that the rabies was raging over the 5 provinces of the island and that dogs were essentially the vector of the virus [9]. We report here the result of the last 6 years of the laboratory surveillance (2005–2010) carried out exclusively by the national authorized laboratory for rabies diagnostic (NLR) at the Institut Pasteur from Madagascar.

## 2. Materials and Methods

### 2.1. Samples

Animal samples tested routinely for rabies consisted of brain, head, or corpse of terrestrial nonflying mammals sent by veterinarians, animal health officers and technicians, animal owners, or persons (or relatives) exposed to these animals. Human samples consisted of postmortem brain biopsies or postmortem skin biopsies taken from the nape of the neck, sent generally at +4°C by hospital staff. Upon reception at the NLR, brain biopsies were kept at +4°C and processed within 48 h. Skin biopsies were kept at −80°C till processing.

Furthermore, samples collected from bats were also tested. They were obtained during a survey looking for virus associated to bats. Samples consisted of sera, blood clots, and pharyngeal swabs kept in viral transport medium (VTM). They were sent within 12 hours to the laboratory and then stored at −80°C at their arrival. When the field was far from the laboratory, they were stored in liquid nitrogen and then transported to the laboratory. When tested, each clot was grinded at a 1 : 10 dilution in cell culture medium (DMEM) containing 30% foetal calf serum and centrifuged at 3,000 rpm for 10 min at +4°C. Then pools of up to 10 supernatants or 10 pharyngeal swabs VTM were constituted before testing.

### 2.2. RABV Antigen Detection

Rabies nucleocapsid detection was performed by fluorescent antibody test (FAT) using rabbit IgG against RABV nucleocapsid (Bio-Rad, Marnes-la-Coquette, France) and performed on the brain postmortem biopsy as the standard [10].

### 2.3. RABV RNA Detection

RNA was extracted from skin biopsies according to the procedure described by Dacheux and colleagues [11]. RNA was extracted also from pools of bats blood clots supernatants or bats pharyngeal swabs VTM using TRIzol LS (Invitrogen, Carlsbad, Calif, USA) and from brain biopsies using TRIzol (Invitrogen, Carlsbad, Calif, USA), as recommended by the manufacturer.

Lyssavirus RNA detection was performed using a reverse transcription and a heminested PCR targeting a conserved region of the polymerase genes of lyssaviruses [11].

### 2.4. RABV Isolation

Virus isolation was performed to confirm the negative result of the rabies virus antigen detection in animal samples tested routinely for rabies. From 2005 through 2007, virus isolation was performed in newborn mice [10], then isolation was performed in cell cultures (Murina neuroblastoma cell line) [12].

Virus isolation in new-born mice was also used for the samples collected from bats.

### 2.5. Detection of Antibodies against Lyssaviruses

Antibodies against RABV, Lagos Bat Virus (LBV), European Bat Lyssavirus type 1 (EBLV-1), EBLV-2, Mokola virus (MOKV), and Australian Bat Lyssavirus (ABLV) were detected in bat sera using lyssavirus rapid fluorescent focus inhibition test [13].

## 3. Results

### 3.1. Rabies Virus Detection in Human and Domestic or Tame Wild Animal Samples

During the 6-year period, the NLR received 461 specimens, 450 from animals and 11 from humans. Most of the 450 animal samples were from domestic carnivorous (*n* = 409, 90.9%), including dogs (*n* = 353, 78.4%) and cats (*n* = 56, 12.4%). We noticed that lemurs, an endemic primate from Madagascar, counted for 2% of the animal samples. All lemurs sampled were reared as pets. Brain was available for all animals. Eleven human suspected rabies cases were also laboratory investigated. Human samples consisted of skin biopsy for 6 cases, brain for 4 cases, and cerebrospinal fluid for one case. Fourteen samples were inadequate and could not be tested, mostly because of inadequate storage (Table 1).

**Figure 1:**
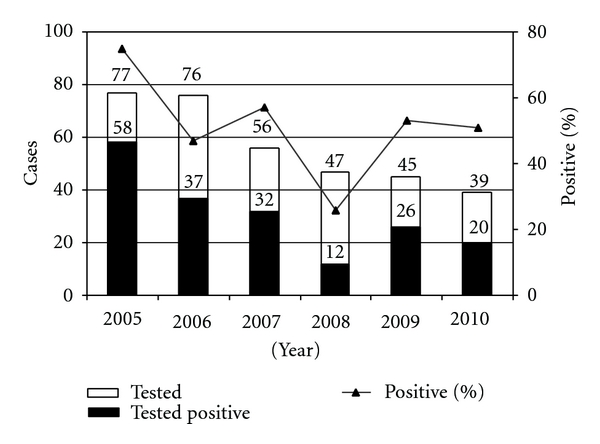
Rabies laboratory diagnostic in dogs, Madagascar, 2005–2010.

Half of the 437 animal specimens tested (all brains) were found positive using FAT. All the samples from lemurs were tested negative. Cattle and pigs, not frequently sampled, were often found positive. More than half of the dogs tested were found infected (Table 1). This percentage varied across the period from 26% (12/47) to 75% (58/77) (Figure 1). When comparing some characteristics of confirmed rabid dogs and RABV noninfected dogs sampled from 2006 through 2010, the positive predictive value was highest for dogs suspected of rabies-clinical disease or unusual spontaneous attack 60.6% (95% CI 53.6%–67.7%), for dogs responsible for bite 50.9% (95% CI 44.3%–57.5%), or for dogs less than 4 years old 57.3% (95% CI 48.9%–65.8%) (Table 2). Nine of the 10 human cases samples tested were found positive (Table 1). The sample tested negative was one skin biopsy.

**Figure 2:**
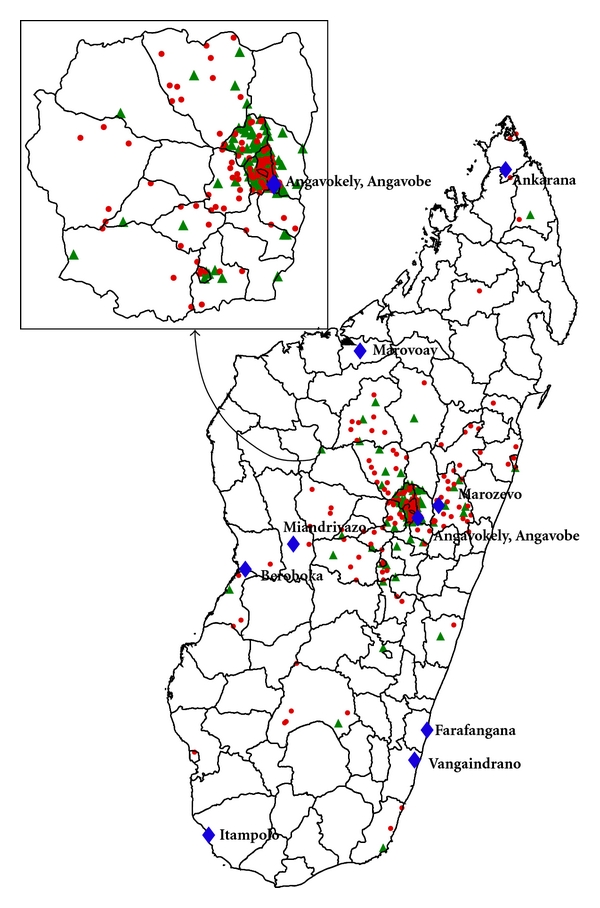
Distribution of the human and nonflying animal samples tested negative (green-filled triangle) and positive (red-filled circle) for rabies, and sites of bats sampling (blue-filled diamond) in Madagascar, 2005–2010.

During the 6-year period, the 447 samples tested were received from 38 of the 111 administrative districts of Madagascar. Most of these samples (365; 82%) were received from Antananarivo province. Rabies circulation was confirmed in 34 of the 38 districts (Figure 2). The virus was present in the capital city of Antananarivo (59 infected animals among 155 tested). Rabies circulation was not detected in 4 of the 38 districts sampled. However, very few samples were received from them (6 samples from one district and 1 sample each from the 3 others).

### 3.2. Lyssavirus and Antibodies against Lyssavirus Detection in Wild Animal Samples

Brain samples from only two wild terrestrial nonflying mammals were received: one fossa (*Cryptoprocta ferox*), the largest mammalian carnivore of Madagascar, and one roof rat (*Rattus rattus*). They tested negative.

A large collection of samples obtained from insectivorous and frugivorous bats were also tested (Table 3). They were collected during (i) a transversal survey looking for henipavirus carried out in 2004 and 2005 in Madagascar [14] and (ii) a longitudinal survey carried out from 2005 to 2009 in Angavobe and Angavokely caves that host Malagasy straw-colored fruit bat (*Eidolon dupreanum*) (Figure 2). No lyssavirus RNAs were detected in these blood samples and oral swabs. No lyssavirus isolates were obtained from all these samples in new-born mice.

Sera from 28 Malagasy flying foxes (*Pteropus rufus*) and from 50 Malagasy straw-colored fruit bats *(Eidolon dupreanum)* were tested for antibodies against lyssaviruses. Antibodies against EBLV-1 and LBV were detected in five and one Malagasy flying fox, respectively. Antibodies against LBV were detected in 12 Malagasy straw-colored fruit bats (24%), titers ranging from 35.2 to 65. No antibodies were detected against MOKV, EBLV-2, and ABLV.

## 4. Discussion

Despite the introduction a century ago of the rabies vaccine in Madagascar, the recurrent positive laboratory diagnostic of rabies in dogs suggests that this zoonotic disease remains endemic in the island (Figure 1). The percentage of dogs detected infected by RABV along the 2005–2010 period (54%; 185/341) was in the same range of the one observed during the 1959–1991 period (57%; 1416/2475) [9]. Dogs remain probably the principal vectors of RABV in the island. RABV strains associated to dogs in Madagascar were shown to belong to the cosmopolitan lineage [15, 16]. There was an evidence of RABV circulation in Antananarivo, the capital city. Antananarivo had, in 2007-2008, a density of dogs higher than many other urban areas in Africa, and the dog population was unrestricted and inadequately vaccinated against rabies, this characteristic favouring probably the dissemination of the virus [17]. This situation is probably not limited to the capital city in Madagascar and may explain the rabies endemic situation in the island.

Several endemic or (few) introduced carnivorous mammals (Families Viverridae and Herpestidae) are present in Madagascar [18]. So far, very few suspected animals from these species have been tested. One rabid confirmed human case was bitten by a fossa (*Cryptoprocta ferox*) in Ihosy district, in 2007, and the strain obtained from this case was confirmed as a lyssavirus of the species RABV, phylogenetically closely related to those circulating in Malagasy dogs (data not shown). Consequently, the question of a possible vector in the wild terrestrial carnivorous mammals remains unanswered. This question is of importance considering a rabies control programme targeting the eradication of the rabies in the island. 

Our extensive survey in bats failed to detect any lyssavirus associated to these mammals. The molecular technique we used to detect lyssaviruses was demonstrated to be sensitive, reproducible, and repeatable [11]. Furthermore, virus isolation on new-born mice was considered sensitive as we isolated several viruses from the bats specimens, like Ife virus from the Malagasy straw-colored fruit bat (*Eidolon dupreanum*) and Dakar bat virus from the Peters's wrinkle-lipped bat (*Mormopterus jugularis*) (unpublished data). Low prevalence of active infection (detection of virus) has been observed in North American and European bats colonies (0.1 to 2.9%), especially in clinically normal bats [19]. Because we sampled clinically normal bats and because our sampling size per site and per species was for the most about 100 animals (except for the site of the followup where we sampled about 750 animals), our negative results in detecting a lyssavirus are consequently not so surprising. Lyssavirus detection was also negative in brains sampled in 1987 and 1988 in Madagascar, from 59 little free-tailed bats (*Chaerephon pumilus*) [20]. Interestingly, we got serological evidence that lyssaviruses have circulated among Malagasy bats. The lyssavirus LBV has been isolated from the African straw-colored fruit bat (*Eidolon helvum*), the second of the two species in this African genus in various countries of Africa [21]. We isolated Ife virus and an alphaherpesvirus from the Malagasy straw-colored fruit bat [22]. These two viral species have also been detected from African straw-colored fruit bat [22, 23]. Therefore, we highly suspected the presence of LBV in Madagascar. Consequently, postexposure rabies vaccination should be provided after an exposure to Malagasy bats. However, people should keep in mind that rabies vaccine is less efficient against lyssavirus belonging to the phylogroup 2, including LBV [24].

We recently showed that a heminested PCR targeting a conserved region of the polymerase genes of lyssaviruses and applied to antemortem or postmortem skin biopsy (a specimen easier to collect than a piece of brain) was a successful procedure to perform rabies diagnostic [11]. We raised centres for postexposure prophylaxis staffs awareness of the performance of this procedure. Since that period (2008), we received postmortem skin biopsies from rabies-suspected cases, some of them coming far from Antananarivo, like Taolagnaro, on the south coast of the country (data not shown). Rabies infection was confirmed in 5 of these 6 cases. These samples easy to perform and to ship to the laboratory should be more promoted among health care personnel through Madagascar, to have a better idea of the prevalence of rabies in humans. Furthermore, this procedure should be also tested on carnivorous mammals, considering the sampling of skin carrying vibrissae (rich in nerve endings surrounding the base of these hairs). This method could help avoiding contamination of people sampling these animals by rabies virus-containing biological fluids and promote the sampling of rabies-suspected animals.

So far, for economic reasons, there are rabies postexposure prophylaxis centres in only 26 of the 111 administrative districts of Madagascar. We received samples of rabies-suspected cases from only 13 of them, and rabies virus circulation was confirmed in all of them. There is a need to confirm repeatedly its circulation in all of these 26 districts, especially in two islands (Nosy Be and Sainte Marie), where there is no recent report of rabid animals. Sampling should be promoted in the 13 other districts to evaluate the pertinence of these centres.

## 5. Conclusion

More than a century after the introduction of the vaccine against rabies in Madagascar, rabies remains endemic in the island. So far, preventing human rabies through dog rabies control and eventual elimination has been limited to local initiative. Madagascar, like other countries, is facing numerous public health issues. Because of the low incomes of the country and the lack of epidemiological data, this disease has not been prioritized, and a control program could not reasonably start. However, Madagascar is an island, and the elimination of rabies and its sustainability should be facilitated by the limited risk of introduction of rabid animals Therefore, the collection of such data (human and animal surveillance, dog ecology study, animal bites, etc.) should be promoted at first on pilot scale in order to validate the tools used. Afterward, data collection should be expanded to the rest of the country, while a pilot rabies control program (canine vaccination, canine population management, human postexposure prophylaxis, education, information, etc.) should start on pilot sites and then extended to the rest of the country.

## Figures and Tables

**Table 1:** Rabies laboratory diagnostic in human, domestic and tame wild animals, Madagascar, 2005–2010.

Species		Samples	
	Received	inadequate	Tested positive (%)
Human	11	1	9 (90)
Dog	353	12	185 (54)
Cat	56	1	13 (24)
Cattle	26	0	21 (81)
Pig	3	0	2 (67)
Rabbit	2	0	0
Lemur	10	0	0

Total	461	14	229 (51)

**Table 2:** Positive predictive values according to some characteristics of dogs tested for rabies (reported alone), Madagascar, 2006–2010.

Characteristics		Rabies laboratory results		Positive predictive values
		Negative	Positive	(%)
Suspected of rabies (*n* = 257)	Yes	74	114	60.6
	No	59	10	14.5
Responsible for bite (*n* = 256)	Yes	111	115	50.9
	No	21	9	30.0
Less than 4 years old (*n* = 180)	Yes	58	78	57.4
	No	33	11	25.0

**Table 3:** Bats samples tested for lyssavirus, according to the species and the site of capture, Madagascar 2005–2009.

Diet and bat Family	Species	Site of capture	No blood samples	No oral swabs
*Insectivorous*				
Hipposideridae	*Triaenops rufus*	Itampolo	18	0
Vespertilionidae	*Myotis goudoti*	Itampolo	1	0
	*Miniopterus gleni*	Itampolo	1	0
	*Chaerephon pumilus*	Vangaindrano	22	0
Molossidae	*Mops leucostigma*	Farafanga	14	0
		Vangaindrano	17	0
	*Mormopterus jugularis*	Itampolo	19	0
*Frugivorous*				
		Marovoay	130	104
Pteropodidae	*Pteropus rufus*	Marozevo	33	8
		Beroboka	29	0
		Miandrivazo	112	97
		Vangaindrano	38	32
		Angavobe	54	32
		Miandrivazo	2	2
	*Eidolon dupreanum*	2005–2009		
		Roost followup	753	465
		Angavobe and Angavokely		

Total			**1243**	**740**
